# The continuing legacy of nature versus nurture in biolinguistics

**DOI:** 10.3758/s13423-016-1202-7

**Published:** 2017-01-24

**Authors:** Daniel L. Bowling

**Affiliations:** Department of Cognitive Biology, University of Vienna, Universitätsring 1, 1010 Vienna, Austria

**Keywords:** Language, Genetics, Development, Nature vs. Nurture

## Abstract

Theories of language evolution that separate biological and cultural contributions perpetuate a false dichotomy between nature and nurture. The explanatory power of future theories will depend on acknowledging the reality of gene–culture interaction and how it makes language possible.

Kirby’s theory puts culture at the heart of language evolution. His thesis is that linguistic structure is best explained as a result of cultural transmission rather than natural selection (Kirby, 2016). He does not exclude biological constraints completely, however, arguing that human predispositions to share our inner thoughts with each other and learn new signals were both critical in setting the stage for the emergence of linguistic structure through culture. On the surface then, Kirby’s account appears to integrate culture with biology, reflecting the modern consensus that explanations cast in terms of nature versus nurture pose a false dichotomy (Bateson, 2002; Gopnik, 2014; Ridley, 2003).

There is an important sense, however, in which Kirby’s theory fails to leave the nature–nurture dichotomy behind. His identification of constraints as either cultural or biological assumes fundamentally that they are separate causal forces. This is a problem, not only because culture is itself a biological phenomenon, but because interactions between DNA and the environment are bidirectional and ubiquitous (Fisher, 2006; Goldberg, Allis, & Bernstein, 2007; Kanherkar, Bhatia-Dey, & Csoka, 2014). This is particularly relevant for linguistic structure, which probably requires extensive interactions between genome and the environment to properly develop (Johnson & Newport, 1989; Werker & Hensch, 2014).

Although many details remain obscure, classic examples of experience-dependent development in other domains like vision strongly suggest that during the cascade of critical and sensitive periods that characterize language learning (Werker & Hensch, 2014), exposure to the right language input at the right times is required for the local expression of certain genes, whose products (e.g., transcription factors, proteolytic enzymes, and neurotrophic factors) and their interactions are in turn required for subsequent input to effect the appropriate neural modifications (Borrelli, Nestler, Allis, & Sassone-Corsi, 2008; Hensch, 2004, 2005; Werker & Hensch, 2014). Given that specific gene-culture interactions are likely to be critical for language development, models that pin high-level linguistic features like structure primarily on culture or biology are unlikely to explain the origins of language.

In historical context, however, Kirby’s emphasis on culture serves as an important counterweight to the widespread view that linguistic structure must be explained by specific causal modifications to the genome (Bolhuis, Tattersall, Chomsky, & Berwick, 2014; Klein & Edgar, 2002; Pinker & Bloom, 1990), a theory that is even more subject to criticism for failing to recognize gene-culture interactions (Fisher & Ridley, 2013; Laland, Odling-Smee, & Myles, 2010). From this perspective, Kirby et al.’s demonstration that cultural transmission, modeled using iterated learning in the presence of an informational bottleneck, can turn a holistic system into a compositional one expands the set of explanatory tools we can use to think about language evolution, and provides a warning against abstract (and probably false) assumptions about the genetic foundations of language. To paraphrase one geneticist’s thoughts on the topic: genes do not encode specific behaviors, cognitive processes, or even neural circuits, they make proteins that interact in complex, environmentally modulated networks, to build and maintain brains (Fisher, 2006, 2016). A final positive point is that even though Kirby’s Bayesian models falsely separate genes from learning, the inclusion and formalization of both is a clear step forward.

In sum, modern theories of language evolution that combine cultural and biological constraints still have work to do before they can leave the nature–nurture dichotomy fully behind. It seems likely that the explanatory power of future theories will ultimately depend on coming to grips with the molecular and neural details of gene–culture interactions and how they make language possible.

## References

[CR1] Bateson, P. (2002). The corpse of a wearisome debate. *Science, 297,* 64–65.

[CR2] Bolhuis, J. J., Tattersall, I., Chomsky, N., & Berwick, R. C. (2014). How could language have evolved? *PLoS Biology, 12*(8), 1–6. doi:10.1371/journal.pbio.1001934 10.1371/journal.pbio.1001934 PMC4144795 25157536

[CR3] Borrelli, E., Nestler, E. J., Allis, C. D., & Sassone-Corsi, P. (2008). Decoding the epigenetic language of neuronal plasticity. *Neuron, 60*(6), 961–974. doi:10.1016/j.neuron.2008.10.012 10.1016/j.neuron.2008.10.012 PMC2737473 19109904

[CR4] Fisher, S. E. (2006). Tangled webs: Tracing the connections between genes and cognition. *Cognition, 101*(2), 270–297. doi:10.1016/j.cognition.2006.04.004 10.1016/j.cognition.2006.04.004 16764847

[CR5] Fisher, S. E. (2016). Evolution of language: Lessons from the genome. *Psychonomic Bulletin & Review*, 1–7. doi: 10.3758/s13423-016-1112-8 10.3758/s13423-016-1112-8 PMC5325862 27432000

[CR6] Fisher, S. E., & Ridley, M. (2013). Culture, genes, and the human revolution. *Science, 340*(6135), 929–930. doi:10.1126/science.1236171 10.1126/science.1236171 23704558

[CR7] Goldberg, A. D., Allis, C. D., & Bernstein, E. (2007). Epigenetics: A landscape takes shape. *Cell, 128*(4), 635–638. doi:10.1016/j.cell.2007.02.006 10.1016/j.cell.2007.02.006 17320500

[CR8] Gopnik, A. (2014). Time to retire the simplicity of nature vs. nurture. *The Wall Street Journal*. Retrieved from http://www.wsj.com/articles/SB10001424052702304302704579334954138196792

[CR9] Hensch, T. K. (2004). Critical period regulation. *Annual Review of Neuroscience, 27*(1), 549–579. doi:10.1146/annurev.neuro.27.070203.144327 10.1146/annurev.neuro.27.070203.144327 15217343

[CR10] Hensch, T. K. (2005). Critical period plasticity in local cortical circuits. *Nature Reviews. Neuroscience, 6*(11), 877–888. doi:10.1038/nrn1787 10.1038/nrn1787 16261181

[CR11] Johnson, J. J. S., & Newport, E. L. (1989). Critical period effects in second language learning: The influence of maturational state on the acquisition of English as a second language. *Cognitive Psychology, 21*(1), 60–99. doi:10.1016/0010-0285(89)90003-0 10.1016/0010-0285(89)90003-0 2920538

[CR12] Kanherkar, R. R., Bhatia-Dey, N., & Csoka, A. B. (2014). Epigenetics across the human lifespan. *Frontiers in Cell and Developmental Biology, 2,* 49. doi:10.3389/fcell.2014.00049 10.3389/fcell.2014.00049 PMC4207041 25364756

[CR13] Kirby, S. (2016). Culture and biology in the origins of linguistic structure. *Psychonomic Bulletin & Review*. 10.3758/s13423-016-1166-7 PMC5325872 28120320

[CR14] Klein RG, Edgar B (2002). The dawn of human culture.

[CR15] Laland, K. N., Odling-Smee, J., & Myles, S. (2010). How culture shaped the human genome: Bringing genetics and the human sciences together. *Nature Reviews Genetics, 11*(2), 137–148. doi:10.1038/nrg2734 10.1038/nrg2734 20084086

[CR16] Pinker, S., & Bloom, P. (1990). Natural language and natural selection. *Behavioral and Brain Sciences, 13*(4), 707–784.

[CR17] Ridley M (2003). Nature via nurture: Genes, experience, and what makes us human.

[CR18] Werker, J. F., & Hensch, T. K. (2014). Critical periods in speech perception: New directions. *Annual Review of Psychology, 66,* 173–196. doi:10.1146/annurev-psych-010814-015104 10.1146/annurev-psych-010814-015104 25251488

